# Cardio-cerebrovascular complications in COVID-19 patients: A retrospective cohort study

**DOI:** 10.3389/fmed.2022.1045274

**Published:** 2022-12-22

**Authors:** Kwan Hong, Trishna Kisiju, Jeehyun Kim, Byung Chul Chun

**Affiliations:** ^1^Department of Public Health, Korea University Graduate School, Seoul, Republic of Korea; ^2^Department of Preventive Medicine, Korea University College of Medicine, Seoul, Republic of Korea; ^3^Department of Healthcare Sciences, Graduate School, Transdisciplinary Major in Learning Health Systems, Korea University, Seoul, Republic of Korea

**Keywords:** COVID-19, SARS-CoV-2, post-COVID conditions, acute cardiovascular complications, acute cerebrovascular complications, retrospective cohort study

## Abstract

**Background:**

Recent studies have highlighted the cardio-cerebrovascular manifestations of coronavirus disease 2019 (COVID-19).

**Objective:**

This study aimed to analyze the likelihood of cardiovascular and cerebrovascular manifestations among patients with COVID-19-positive individuals in South Korea.

**Methods:**

A cohort database for COVID-19 from the National Health Insurance Service was used which included patients diagnosed with COVID-19 between January 1 and June 4, 2020. Individuals who tested COVID-19 positive, notwithstanding the severity of the disease, were designated as cases. COVID-19- negative individuals were used as controls for the study. The exclusion criteria included people who had a history of cardiovascular and cerebrovascular diseases between 2015 and 2019. A new diagnosis of cardiovascular and cerebrovascular complications was considered the primary endpoint. The adjusted incidence rate ratio (IRR) of development of complications was estimated using log-link Poisson regression. The model was adjusted at two levels, the first one included age and sex while the second included age, sex, residence area, and level of income. The hazard ratio (HR) was estimated using Cox-proportional hazard regression analysis while adjusting for all demographic variables and covariates.

**Results:**

Significant results were obtained for acute conditions, such as ischemic heart disease and cerebral hemorrhage. The IRR of COVID-19- positive individuals compared with that of controls for the diagnosis of ischemic heart disease was 1.78 (1.57–2.02; 95% confidence interval [CI]) when adjusted for age and sex. HR was calculated as 3.02 (2.19–4.17; 95% CI) after adjusting for the covariates. In case of cerebral hemorrhage, the adjusted IRR was 2.06 (1.25–3.40; 95% CI) and the adjusted HR was 4.08 (0.90–19.19; 95% CI).

**Conclusion:**

The findings of our study suggest that COVID-19 infection can be a significant risk factor for acute cardiovascular complications, such as ischemic heart disease and acute cerebrovascular complications, such as cerebral infarction, after properly adjusting for covariates.

## 1 Introduction

As of March 31, 2022, total coronavirus disease 2019 (COVID-19) cases have soared to over 486 million worldwide with over 6.16 million deaths and over 421 million survivors (1). Concerns regarding COVID-19 inducing cardiovascular and cerebrovascular manifestations, in addition to the clinical presentation of respiratory failure have increased (2). Complications in patients with COVID-19 include cardio-cerebrovascular conditions, such as shock, acute cardiac injury, and arrhythmias (3).

Angiotensin-converting enzyme 2 (ACE 2) acts as a gateway for the entry of the severe acute respiratory syndrome coronavirus 2 (SARS-CoV-2) into the host cell (4). Direct cardiovascular manifestations in COVID-19-positive individuals, such as myocarditis, heart failure, and arrhythmias are based on the pathophysiology of the renin-angiotensin system (RAS) axes, while the indirect effects such as thromboembolism and metabolic disorders are caused by the cytokine storm (5, 6). A cytokine storm is thought to result in myocardial injury, that involves elevated production of interleukin-6 (IL-6), ferritin, D-dimer, lactate dehydrogenase (LDH), etc. (7). Many studies have suggested that patients with pre-existing cardiovascular diseases experience severe COVID-19 outcomes (8–11) while others have reported that COVID-19 itself can be a risk factor for cardiovascular complications (12). The pathogenesis of cerebrovascular diseases in patients with COVID-19, is thought to be mediated through endothelial dysfunction caused by infection of endothelial cells and initiation of a coagulation cascade resulting in a state of hypercoagulability that can contribute to various thromboembolic events (13).

Several previous studies have demonstrated COVID-19 to be an important risk factor for acute myocardial infarction and acute ischemic stroke (11). However, some studies have shown that cardiovascular diseases, such as ischemic stroke are not positively associated with COVID-19 (14). The studies that have determined the acute cardio-cerebrovascular manifestations of COVID-19 using a control group are limited and have a restricted sample size. Furthermore, there is a need for nationally representative data to effectively identify cardiovascular and cerebrovascular insults caused by COVID-19. Thus, with the use of national representative data, this study aimed to determine the risk of acute cardio-cerebrovascular manifestations in the COVID-19 positive patients in South Korea.

## 2 Materials and methods

### 2.1 Study design

COVID-19 cohort database provided by the National Health Insurance Service (NHIS) was used for data collection. The database consisted of NHIS subscribers. The database was formulated with its sole purpose of application in medical research, as a collaboration of the NHIS with the Korea Centers for Disease Control and Prevention (KCDC).

The KCDC provided information on COVID-19 patients and controls, who were diagnosed from January 1, 2020, to June 4, 2020, to the NHIS COVID-19 cohort database. The NHIS-COVID-19 cohort database was opened per research team for about 5 days. The database encompasses information on the cases and controls, including demographic characteristics, official COVID-19 diagnosis confirmation date, medical history of the subjects, results of medical checkups, treatment results and demise. Diagnoses were classified based on International Classification of Diseases (ICD)-10 codes. The database excluded information regarding individuals with incomplete medical records and foreign patients with COVID-19 (15).

### 2.2 Study participants

**FIGURE 1:**
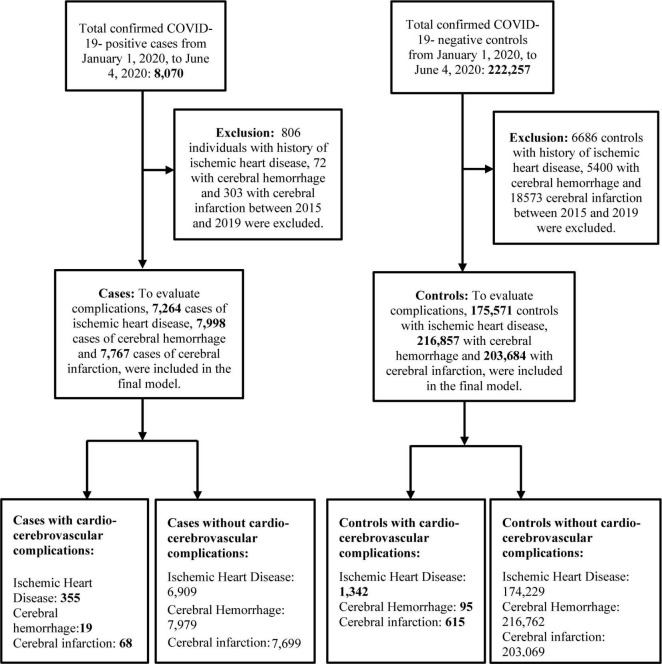
Flowchart of the selection of study participants. Ischemic heart disease was diagnosed with I20–I25, cerebral hemorrhage with I60–I62 and cerebral infarction with I63 in ICD-10-Code. COVID-19, coronovirus disease 2019.

Individuals diagnosed through real-time reverse transcription polymerase chain reaction test (RT-PCR) for SARS-CoV-2 with cycle threshold (Ct) cutoff value of 33.5 were defined as cases (16), while the controls were individuals who tested negative for COVID-19. Individuals with a history of cardiovascular and cerebrovascular diseases between 2015 and 2019 were excluded. Figure 1 shows the flowchart of the selection of study participants.

### 2.3 Statistical analysis

Individuals from the cohort database with history of cardio-cerebrovascular conditions between 2015 and 2019 were excluded to analyze the development of a new cardio-cerebrovascular complication after the diagnosis of COVID-19. The potential confounders designated for cardio-cerebrovascular complications of COVID-19 were age (17, 18), sex (17–20), place of residence (18, 19, 21) and income level (18, 22). Age was classified based on 10 years age groups and the residential area was categorized as Seoul, Gyeonggi-do, Daegu, Gyeongsangbuk-do and others. Similarly, income level had a value ranging from 0 to 20. The secondary potential confounders considered were drinking alcohol frequency (23–25), smoking history (26–28), comorbidities like Diabetes Mellitus (DM) (29, 30), hypertension (31, 32) and dyslipidemia (33, 34), anthropometric variables like body mass index (BMI) (19, 35, 36), glomerular filtration rate (GFR) (37, 38), gamma-glutamyl transferase level (GGT-level) (39, 40), hemoglobin level (Hb-level) (41, 42), height (43) and family history of heart disease (44). The significant secondary potential confounders were adjusted using Cox proportional regression analysis.

Follow-up of the cases started from the date of confirmation of COVID-19 diagnosis. The diagnosis confirmation date, however, might differ from the actual date of diagnosis since the date of the official diagnosis announcement was used. The follow-up of controls started on June 4, 2020 (the final date of diagnosis of COVID-19). The manifestation of cardiovascular and cerebrovascular complications in patients with COVID-19 in the cohort database was regarded as the primary endpoint of the study. This endpoint was evaluated from January 1, 2020, to June 4, 2020.

The demographics of the cases and controls were analyzed through descriptive statistics, independent sample *t*-test and chi-squared test. Log-link Poisson regression analysis and Cox proportional regression analyses were used for sensitivity analysis. Based on the incidence density, a log-link Poisson regression model was used to estimate the adjusted incidence rate ratio (IRR). Adjustment was performed at two levels, one for age and sex, and another for age, sex, residence area and income level. The hazard ratio (HR) was estimated using Cox proportional regression, while adjusting for primary and secondary potential confounders.

SAS Enterprise Guide (version 7.13) was used to conduct all statistical analyses. Statistical significance for the analyses was set at *p* < 0.05. The Institution Review Board (IRB) of Korea University approved the exemption from ethical approval (IRB exemption number: KUIRB-2021-0238-02).

The evaluated cardio-cerebrovascular complications included (a) ischemic heart disease (I20–I25), (b) cerebral hemorrhage (I60–I62) and (c) cerebral infarction (I63).

## 3 Results

This study evaluated 2,30,327 individuals, of which 8,070 were COVID-19 positive patients (cases) and 2,22,257 were controls. The demographic characteristics of the study subjects are described previously (45). The highest number of COVID-19- positive patients was present in the 20–29-year age group (2057/8070; 25.5%). Similarly, the highest number of controls was in the 30–39-year age group (37977/222257; 17.1%). The number of female participants dominated both the cases (4834/8070; 59.9%) and controls (116175/222257; 52.3%). Diabetes was 14.4% in the positive group and 13.5% in the control group, showing no significant difference (*p* = 0.157), and there was no significant difference between the two groups in hypertension (27.4 vs. 27.3%) and hyperlipidemia (10.1 vs. 9.6%, respectively) (45). The final model included different number of cases and controls depending on the type of the disease under study. Table 1 shows the number of new cardio-cerebrovascular complications that occurred in patients and controls during the follow-up.

**TABLE 1:** New diagnosis of cardio-cerebrovascular complications among the COVID-19- positive cases and controls.

	COVID-19 positive patients (*n* = 8,070)	Controls (*n* = 2,22,257)	*p*-value*
New diagnosis of ischemic heart disease			<0.001
Yes	355/7,264 (4.9%)	1,342/1,75,571 (0.76%)	
No	6,909/7,264 (95.1%)	1,74,229/1,75,571 (99.24%)	
New diagnosis of cerebral hemorrhage			<0.001
Yes	19/7,998 (0.23%)	95/2,16,857 (0.04%)	
No	7,979/7,998 (99.77%)	2,16,762/2,16,857 (99.96%)	
New diagnosis of cerebral infarction			< 0.001
Yes	68/7,767 (0.88%)	615/2,03,684 (0.3%)	
No	7,699/7,767 (99.12%)	2,03,069/2,03,684 (99.7%)	

*p-values were calculated using chi-squared tests.

a. Ischemic heart disease (I20–I25): A total of 7,264 cases and 1,75,571 controls were included in the final model after excluding 806 patients and 6,686 controls with a prior history of ischemic heart disease. A total of 355/7,264 (4.9%) patients and 1,342/1,75,571 (0.76%) controls were newly diagnosed with ischemic heart disease.
b. Cerebral hemorrhage (I60–I62): After the exclusion of 72 cases and 5,400 controls with a history of cerebral hemorrhage, our final model included 7,998 cases and 2,16,857 controls. A total of 19/7,998 (0.23%) cases and 95/2,16,857 (0.04%) controls were newly diagnosed with cerebral hemorrhage.
c. Cerebral infarction (I63): A total of 303 cases and 18,573 controls were excluded owing to a history of cerebral infarction. The final analysis of the study included 7,767 cases and 2,03,684 controls. A total of 68/7,767 (0.88%) cases and 615/2,03,684 (0.3%) controls were newly diagnosed with cerebral infarction.

Table 2 depicts the results of the sensitivity analysis, that include adjusted IRR and adjusted HR. Significant results were obtained in case of ischemic heart disease and cerebral hemorrhage. In case of ischemic heart disease, the age and sex adjusted IRR was 1.78 (1.57–2.02) and HR adjusted for all demographic variables was 3.02 (2.19–4.17). In case of cerebral hemorrhage, the age and sex adjusted IRR was 2.06 (1.25–3.40). Survival analyses failed to show a significant association between COVID-19 diagnosis and risk of cerebral infarction.

**TABLE 2:** Results of sensitivity analyses (log-link Poisson regression and Cox proportional hazard regression).

Cardio-cerebrovascular diseases	Starting date for follow-up of the controls (June 4, 2020)
**Ischemic heart disease I20–I25**	
Adjusted IRR (95 CI)*	1.78 (1.57–2.02)
Adjusted IRR (95 CI)^†^	1.60 (1.36–1.85)
Adjusted HR (95 CI)^‡^	3.02 (2.19–4.17)
**Cerebral hemorrhage I60–I62**	
Adjusted IRR (95 CI)*	2.06 (1.25–3.40)
Adjusted IRR (95 CI)^†^	2.02 (1.09–3.73)
Adjusted HR (95 CI)^‡^	4.08 (0.90–19.19)
**Cerebral infarction I63**	
Adjusted IRR (95 CI)*	1.08 (0.84–1.40)
Adjusted IRR (95 CI)^†^	0.96 (0.71–1.30)
Adjusted HR (95 CI)^‡^	1.29 (0.54–3.07)

IRR, incidence rate ratio; HR, hazard ratio. *Adjusted for age and sex. ^†^Adjusted for age, sex, residence area and income level. ^‡^Adjusted for demographic variables, health behaviors, anthropometric values, and covariates.

## 4 Discussion

Since the study participants in the final analysis excluded individuals with a history of cardiovascular diseases, cerebrovascular diseases, and traditional vascular risk factors, our findings suggest that COVID-19 is an important risk factor for acute cardio-cerebrovascular complications, such as ischemic heart disease and cerebral hemorrhage even after adjusting for covariates. This implies that besides the primary pulmonary sequelae of COVID-19, cardio-cerebrovascular symptoms due to the infection can also cause morbidity and mortality. The robustness of the data used in our study is accentuated by the fact that they were nationally representative.

The findings from existing literature strengthen the results of our study. In a self-controlled case series and matched cohort study in Sweden, a significant increase in the risk of developing myocardial infarction, post COVID-19 infection was observed mainly in the first and second weeks of infection (11). A study in Romania reported fatal intracranial hemorrhage in COVID-19- positive patients who were otherwise healthy individuals without any history of cerebrovascular pathology (46). Similarly, in another case report, massive cerebral hemorrhage was observed in a young COVID-19 positive patient (47). Several studies have implied that respiratory infections can contribute to the development of new cardiovascular and cerebrovascular diseases (48–55).

Our study found an insignificant association between COVID-19 and cerebral infarction, although most existing literature suggests a strong association (10, 56–59). However, the findings from a multicenter cross-sectional study conducted in the healthcare system in New York State stated that there was no apparent presentation of stroke among patients with COVID-19 (14).

Existing literature on the increased risk of acute cardiovascular complications in COVID-19-positive individuals suggest that several mechanisms may be responsible. Some of the common mechanisms include:

i Downregulation of ACE2 receptors leading to Renin-angiotensin-aldosterone system (RAAS) dysregulation: Direct acute myocardial injury may result from binding of SARS-CoV-2 to ACE2 because of alteration in the ACE2 signaling pathways (60, 61).
ii Cytokine release syndrome and hemodynamic instability: Acute systemic inflammation and cytokine release syndrome caused by elevated levels of ferritin, Interleukin-6 (IL-6), IL-2, tumor necrosis factor (TNF)-α, C-reactive protein (CRP), D-dimer and high-sensitivity cardiac troponin I leading to multiple organ failure (including the heart) can occur mostly in severe cases of COVID-19 (60, 62–64).
iii Impaired myocardial oxygen demand-supply ratio: Myocardial injury may occur, subjected to hypoxia induced apoptosis of cardiac myocytes (62, 63).
iv Plaque destabilization leading to microthrombus formation: Increased levels of catecholamines, C-reactive proteins, etc. can precipitate plaque rupture that initiates formation of microthrombi and hence lead to acute myocardial infarction (60).
v Prothrombotic state and coagulation disorders leading to micro thrombosis in various organs: COVID-19-associated coagulopathy may manifest myocardial infarction and microvascular obstruction (65).
vi Imbalance in the electrolytes: Electrolyte imbalance like Hypokalemia specially in severe COVID-19 cases, can be linked to arrythmias (63, 66).

Acute cerebrovascular diseases are thought to occur in COVID-19-positive individuals because of multifactorial etiology, some of which include:

i Neurotropism: Evidence suggest that the brain contains some ACE2 receptors, that makes it a potential target of SARS-CoV-2 (67). The route of entry of the virus maybe through hematogenous spread and/or neuronal retrograde routes (68, 69).
ii Endothelial dysfunction (ED): The Angiotensin II system in the brain is linked with multiple functions like brain development and cerebral blood flow (70). Since ACE2 receptors are expressed in endothelial cells of the cerebrovascular system as well, COVID-19 may lead to microbleeds, hemorrhagic lesions and vasogenic edema induced by disruption of the blood brain barrier (71).
iii Hyper coagulopathy: COVID-19 may lead to development of a hypercoagulable state characterized by a prolonged prothrombin time, surge in D-dimers and disseminated intravascular coagulation (DIC) (72).
iv Inflammation: Elevated levels of IL-1β, IL-10, IL-6, TNF-α, etc. induce an inflammatory state and cytokine cascade that can cause intracerebral hemorrhage mostly in severe cases of COVID-19 (73).

Our study has some limitations. There is a possibility that the cardio-cerebrovascular manifestations that occurred in these cases could have occurred during the course of treatment and medications against COVID-19 infection. In addition, they could have developed through COVID-19 related stress since stress-related cardio-cerebrovascular complications are common (74). In addition, the development of cardio-cerebrovascular complications is thought to depend on the severity of COVID-19 infection (75), but the information regarding severity of the infection was unavailable. In addition, for privacy policy, the date of COVID-19 diagnosis used in the database was not the actual date of diagnosis but the official date of announcement of the diagnosis. Nevertheless, since the official announcement date follows the actual date of diagnosis, bias can tend toward null. This study consisted of nationally representative cohort data along with an appropriate control group and suggested that COVID-19 can be an independent risk factor for acute cardio-cerebrovascular diseases, such as ischemic heart disease and cerebral hemorrhage, even after properly adjusting for covariates.

## Data availability statement

The original contributions presented in this study are included in the article/supplementary material, further inquiries can be directed to the corresponding author.

## Ethics statement

The studies involving human participants were reviewed and approved by Institutional Review Board of Korea University. Written informed consent for participation was not required for this study in accordance with the national legislation and the institutional requirements.

## Author contributions

JK, KH, and BC contributed to the conception and design of the work. JK and KH contributed to data acquisition and analysis. TK and KH drafted the manuscript. All authors critically revised the manuscript, contributed to the interpretation of the work, gave final approval, and agreed to be accountable for all aspects of work ensuring integrity and accuracy.
